# Cardiac Arrhythmia and Heart Failure Shortly After Starting Romosozumab for Osteoporosis: A Case-Based Review

**DOI:** 10.7759/cureus.50303

**Published:** 2023-12-11

**Authors:** Khalid A Alnaqbi, Jawaher Al Zeyoudi, Asma K Aljaberi

**Affiliations:** 1 Internal Medicine, College of Medicine and Health Sciences, United Arab Emirates (UAE) University, Al Ain, ARE; 2 Internal Medicine/Rheumatology, Tawam Hospital, Al Ain, ARE; 3 Rheumatology, Sheikh Shakhbout Medical City, Abu Dhabi, ARE; 4 Endocrinology, Tawam Hospital, Al Ain, ARE

**Keywords:** osteoporosis, congestive heart failure, atrial fibrillation (af), adverse drug events, adverse cardiac events, major adverse cardiac and cerebrovascular events (macce), biologic treatment, romosozumab, anti-osteoporosis drugs

## Abstract

Romosozumab is a humanized monoclonal antibody that targets the sclerostin protein, which regulates bone formation and resorption. It is a novel therapy in the treatment of post-menopausal women with osteoporosis. The evidence regarding romosozumab's cardiovascular safety is conflicting. We report the first post-marketing case demonstrating cardiac events (i.e., atrial fibrillation and congestive heart failure) in a female patient with osteoporosis likely triggered by romosozumab. A literature review on romosozumab and cardiovascular disease is discussed extensively. For osteoporotic patients with cardiovascular risk factors (e.g., hypertension, coronary artery disease, and stroke), the benefits of fracture prevention should be weighed against potential cardiovascular risks before prescribing romosozumab. Real-world data on post-marketing surveillance will shed light on the potential safety signals of romosozumab.

## Introduction

Osteoporosis is characterized by reduced bone mass and compromised bone strength, leading to an increased risk of fragility fractures. It poses a significant public health concern, with a global prevalence of 23.1% in women based on 70 studies, including 800,457 women [1]. Using quantitative ultrasound among Emiratis aged 15-85, the prevalence of osteoporosis was 3.1% (3.2% in women, 2.7% in men) in 2016 [2].

One of the most recently approved drugs for osteoporosis is romosozumab, a humanized monoclonal antibody. The Food and Drug Administration (FDA) approved it in April 2019 to treat post-menopausal osteoporosis. It is the first drug for osteoporosis that has a dual effect of increased bone formation and decreased resorption [3]. In the Active-Controlled Fracture Study in Postmenopausal Women With Osteoporosis at High Risk (ARCH) randomized clinical trial (RCT), post-menopausal women with osteoporosis who received romosozumab had a 48% reduced risk of new vertebral fracture and a 38% reduced risk of hip fracture compared with those who took alendronate [4].

## Case presentation

A 78-year-old woman of Arabic origin presented to the emergency department of a tertiary hospital on May 14, 2022, with complaints of four days of shortness of breath, palpitations, and fatigue. Her medical history was significant for osteoporosis, well-controlled type 2 diabetes mellitus, hyperlipidemia, treated esophageal cancer in 2006, ischemic stroke in 2018 with no residual deficits, and stage 2 chronic kidney disease. She also has a history of light-chain monoclonal gammopathy of unknown significance, diagnosed in November 2021. There were no previous documented episodes of heart failure or myocardial infarctions. 

**Figure 1:**
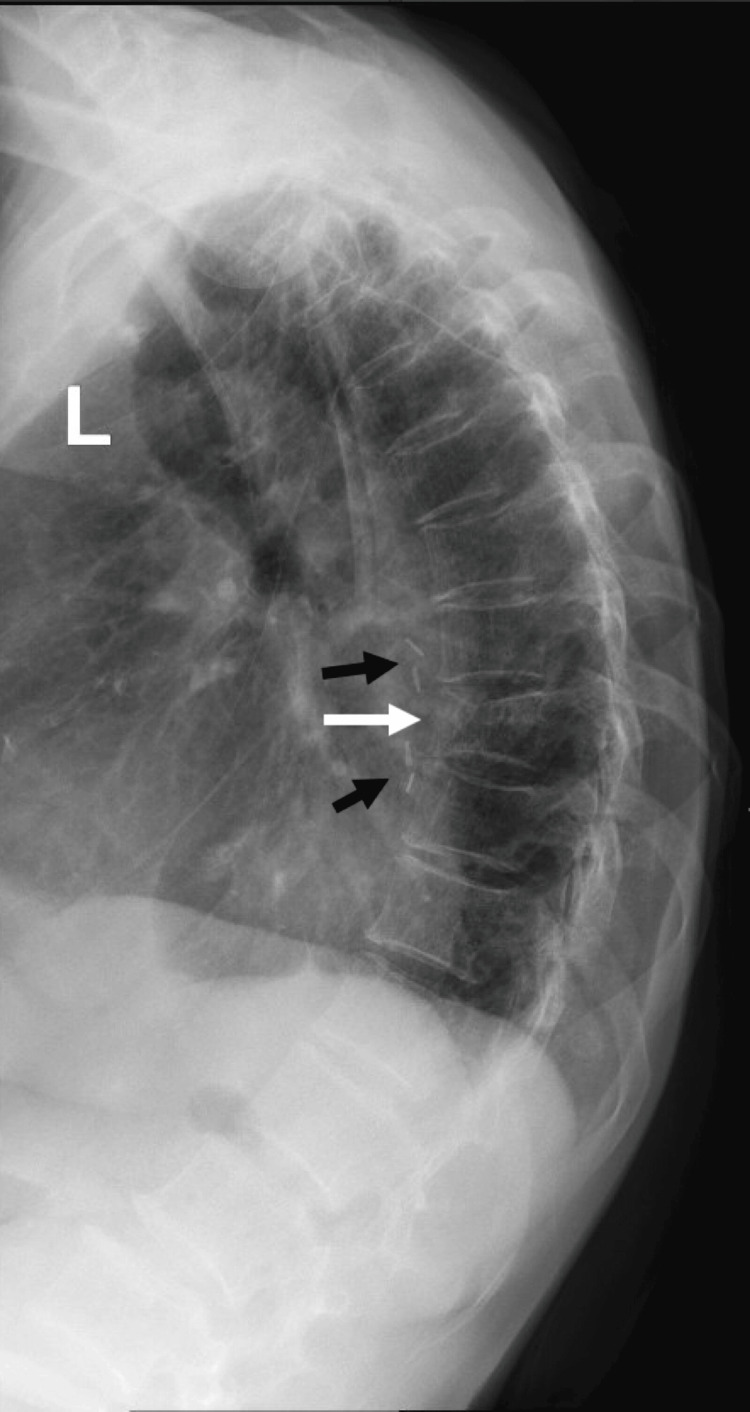
Lateral thoracic spine X-ray showing thoracic kyphosis and compression fracture of T9 vertebral body (white arrow), in addition to surgical clips (black arrow) status post esophagectomy

She was treated with strontium ranelate for her osteoporosis from 2011 to 2014, followed by denosumab from December 2014 to April 2021. Despite this treatment, she sustained a partial compression fracture of the T9 thoracic vertebral body (Figure 1) in August 2021 when the Fracture Risk Assessment Tool (FRAX) indicated a 10-year probability of a major osteoporotic fracture at 8.9% and at 3.0% for hip fracture. Bone mineral density (BMD) parameters of GE Lunar dual-energy X-ray absorptiometry (DXA) machine (GE Medical Systems Ultrasound & Primary Care Diagnostics, Madison, Wisconsin, United States) were as follows: 0.751 g/cm2 for L1-4 (T score: - 3.6), 0.882 g/cm2 for the right femoral neck (T score: - 1.0), 0.874 g/cm2 for the left femoral neck (T score: - 1.1), and 0.576 g/cm2 for the left radius 33% (T score: - 3.4). Her atherosclerotic cardiovascular disease (ASCVD) risk over the next 10 years using the QRISK3 score is 22.8%.

Given the occurrence of the new fracture while on denosumab, her physician switched her treatment to romosozumab 210 mg, administered via monthly subcutaneous injections, which she received three injections in mid-February 2022, mid-March 2022, and mid-April 2022 prior to her presentation to the emergency department. Home medications at the time of hospital presentation included ergocalciferol 50,000 units orally every two weeks, daily calcium 600 mg, aspirin 100 mg, ferrous fumarate 100 mg, atorvastatin 40 mg, and twice-daily metformin 500 mg. 

On physical examination, the patient's pulse was irregularly irregular, with a heart rate of 150 beats per minute. Her blood pressure was 141/50 mmHg, and her oxygen saturation was 92% on room air. She was afebrile and tachypnoeic. Auscultation of her chest revealed bibasilar crackles. Her jugular venous pressure was distended. Cardiac auscultation revealed normal S1 and S2. Due to tachycardia, it was difficult to find a murmur. She did not have lower limb edema. There were equal palpable bilateral pulses, without carotid or femoral bruits. Laboratory tests showed normal white blood cells, liver enzymes, and C-reactive protein and HbA1c of 6.6%. Serum creatinine was 83 umol/L and estimated glomerular filtration rate (eGFR) 59 mL/min/1.73 m^2^.

**Figure 2:**
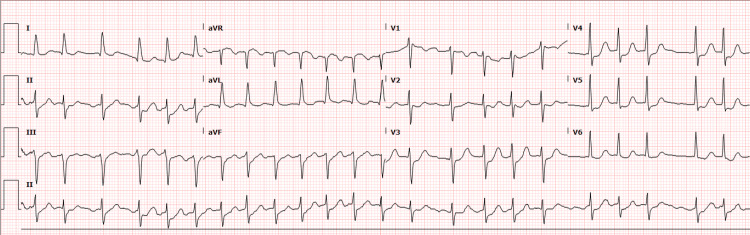
Electrocardiogram of a 78-year-old woman on presentation to our hospital, showing atrial fibrillation

An electrocardiogram showed uncontrolled fast atrial fibrillation with a rapid ventricular response, left-axis deviation, and non-specific ST depression precordial leads, suggesting the possibility of left anterior descending artery disease (Figure 2).

**Figure 3:**
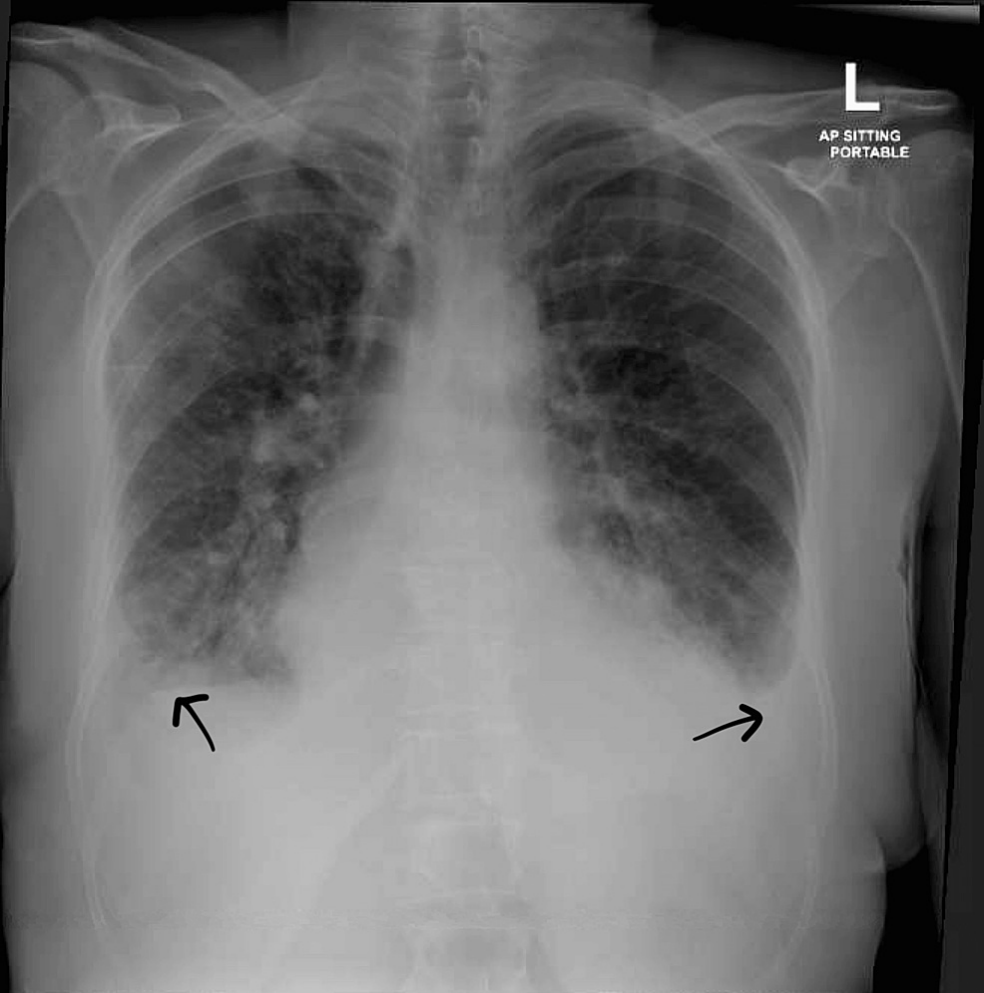
Chest X-ray (anteroposterior view) showed mild bilateral pleural effusion (back arrows) with redistribution of pulmonary vessels

Her chest X-ray showed bilateral pulmonary congestion and mild bilateral pleural effusion (Figure 3).

Urgent transthoracic echocardiography (TTE) showed grade 2 diastolic dysfunction with left ventricular ejection fraction of 55%. It also showed sclerotic aortic valve, with moderate aortic regurgitation. However, assessment was limited due to the tachyarrhythmia.

She was diagnosed with acute pulmonary edema and was started on diuretics and beta-blocker metoprolol. Sestamibi scan excluded myocardial ischemia and showed a preserved left ventricular ejection fraction of 65%. Upon resolution of acute pulmonary edema, she was discharged from the hospital on atorvastatin, edoxaban, metoprolol, metformin, calcium carbonate, ergocalciferol, and ferrous fumarate. Romosozumab was discontinued due to suspicion of a drug-induced adverse event.

During a follow-up clinic visit, she remained asymptomatic. After a discussion with the patient on appropriate anti-osteoporosis medication, she declined taking any medication due to fear of potential side effects. In May 2023, she agreed to restart denosumab as she declined to take a daily subcutaneous anabolic medication.

## Discussion

Romosozumab inhibits the function of a protein called sclerostin [5]. Sclerostin is naturally produced in the bone and acts as an inhibitor of bone formation. By targeting and neutralizing sclerostin, romosozumab effectively promotes bone formation by stimulating the activity of osteoblasts, which are responsible for building new bone tissue [6].

Asadipooya and Weinstock highlighted the role of the Wnt signaling pathway in bone and cardiovascular health. They suggested that romosozumab, which blocks the Wnt inhibitor sclerostin, may increase the risk of cardiovascular adverse events. Bisphosphonate, in combination with romosozumab, was proposed as a potential therapeutic approach to reduce these risks [7]. The proposed mechanism is that inhibiting sclerostin may theoretically promote the formation of vascular calcification and, consequently, atherosclerosis [6]. Zheng et al. investigated the effects of lowering sclerostin levels. The results suggested that lower levels of sclerostin may increase the risk of hypertension, type 2 diabetes mellitus, myocardial infarction, and coronary artery calcification. The study also identified genetic variants associated with sclerostin levels and explored their functional implications [8].

We conducted a literature review in English using PubMed, Web of Science, and Google Scholar databases. Search terms included "romosozumab," "heart failure," "atrial fibrillation," "cardiac events," "myocardial infarction," and "angina." No time limits were applied. We could not identify any case report or case series from the post-marketing data on romosozumab-related cardiac events.

The cardiovascular safety of romosozumab has been a topic of investigation and discussion. The information in the literature is conflicting. Some studies and reports have raised concerns about potential cardiovascular risks associated with its use, while others have not found a significant increase. 

A pharmacovigilance analysis of the cardiovascular safety profile of romosozumab using data from the FDA Adverse Event Reporting System (FAERS) found a potential signal for elevated major cardiovascular events (MACE), particularly in Japan compared to the United States or other countries. The patient profile typically included older men (when the drug did not restrict the inclusion of men in Japan before September 2019) who were on cardiac medications, such as calcium channel blockers, angiotensin II receptor blockers, and anti-platelets. This profile suggests the presence of cardiovascular risk factors [9].

However, the ARCH trial revealed that 2.5% of the patients in the romosozumab group experienced severe cardiovascular events such as myocardial infarction, heart failure, and strokes, while 1.9% of those randomized to alendronate experienced similar events. The rate of cardiovascular death was similar between romosozumab and alendronate groups: 0.8% and 0.6%, respectively [4]. Furthermore, in year 1 of the much larger RCT (Fracture Study in Postmenopausal Women with Osteoporosis (FRAME)), cardiovascular event rates were almost identical in the romosozumab and placebo group: 0.5% vs. 0.4%, respectively [10]. BRIDGE was an RCT that involved 245 men with osteoporosis and found a small increase in serious cardiovascular events in the romosozumab group compared to placebo (4.9% vs. 2.5%, respectively) particularly in those with existing cardiovascular risks [11]. Furthermore, another recent systematic review and meta-analysis revealed that romosozumab did not increase or reduce specific cardiovascular outcomes in primary osteoporosis, including myocardial infarction, atrial fibrillation, or heart failure [12]. Similarly, another systematic review and meta-analysis revealed comparable rates of total and severe adverse events between romosozumab and the control group [13].

These conflicting findings highlight the need for further research and larger-scale studies to better understand the potential cardiovascular risks of romosozumab. Furthermore, none of the RCTs conducted so far on romosozumab have included patients from Arab countries. Therefore, a prospective study in the Arab countries will provide real-world evidence of the efficacy and safety signals of anti-osteoporotic medications. 

While our patient has risk factors that might contribute to the development of atrial fibrillation and congestive heart failure (e.g., age, diabetes mellitus, chronic kidney disease), the temporal relationship coinciding with romosozumab intake raises a strong suspicion that the drug may have triggered cardiac events.

## Conclusions

To our knowledge, this is the first post-marketing case demonstrating cardiac events in a woman with osteoporosis shortly following romosozumab intake. Although romosozumab is considered a breakthrough drug in osteoporosis treatment, our case emphasizes the need to tailor its prescription to individual patients' profiles. In patients with a history of cardiovascular disease or significant risk factors, the benefits of fracture prevention with romosozumab should be weighed against potential cardiovascular adverse events. Close monitoring is essential to minimize these potential adverse events. Real-world data from large population-based electronic medical records or administrative claims data will shed light on the efficacy and potential safety signals of anti-osteoporotic medications particularly in certain ethnic populations not recruited in the original RCTs.

## References

[1] Salari N, Ghasemi H, Mohammadi L, Behzadi MH, Rabieenia E, Shohaimi S, Mohammadi M (2021). The global prevalence of osteoporosis in the world: a comprehensive systematic review and meta-analysis. J Orthop Surg Res.

[2] Al Saleh J, Sayed ME, Monsef N, Darwish E (2016). The prevalence and the determinants of musculoskeletal diseases in Emiratis attending primary health care clinics in Dubai. Oman Med J.

[3] (2023). Romosozumab (Evenity): CADTH Reimbursement Review. Therapeutic area: Osteoporosis [Internet].

[4] Saag KG, Petersen J, Brandi ML (2017). Romosozumab or alendronate for fracture prevention in women with osteoporosis. N Engl J Med.

[5] Miller SA, St Onge EL, Whalen KL (2021). Romosozumab: a novel agent in the treatment for postmenopausal osteoporosis. J Pharm Technol.

[6] Gay A, Towler DA (2017). Wnt signaling in cardiovascular disease: opportunities and challenges. Curr Opin Lipidol.

[7] Asadipooya K, Weinstock A (2019). Cardiovascular outcomes of romosozumab and protective role of alendronate. Arterioscler Thromb Vasc Biol.

[8] Zheng J, Wheeler E, Pietzner M (2023). Lowering of circulating sclerostin may increase risk of atherosclerosis and its risk factors: evidence from a genome-wide association meta-analysis followed by Mendelian randomization. Arthritis Rheumatol.

[9] Vestergaard Kvist A, Faruque J, Vallejo-Yagüe E, Weiler S, Winter EM, Burden AM (2021). Cardiovascular safety profile of romosozumab: a pharmacovigilance analysis of the US Food and Drug Administration Adverse Event Reporting System (FAERS). J Clin Med.

[10] Cosman F, Crittenden DB, Adachi JD (2016). Romosozumab treatment in postmenopausal women with osteoporosis. N Engl J Med.

[11] Lewiecki EM, Blicharski T, Goemaere S (2018). A phase III randomized placebo-controlled trial to evaluate efficacy and safety of romosozumab in men with osteoporosis. J Clin Endocrinol Metab.

[12] Lv F, Cai X, Yang W, Gao L, Chen L, Wu J, Ji L (2020). Denosumab or romosozumab therapy and risk of cardiovascular events in patients with primary osteoporosis: systematic review and meta- analysis. Bone.

[13] Singh S, Dutta S, Khasbage S, Kumar T, Sachin J, Sharma J, Varthya SB (2022). A systematic review and meta-analysis of efficacy and safety of romosozumab in postmenopausal osteoporosis. Osteoporos Int.

