# Correction: Cardiac Arrhythmia and Heart Failure Shortly After Starting Romosozumab for Osteoporosis: A Case-Based Review

**DOI:** 10.7759/cureus.c148

**Published:** 2023-12-20

**Authors:** Khalid A Alnaqbi, Jawaher Al Zeyoudi, Asma K Aljaberi

**Affiliations:** 1 Internal Medicine, College of Medicine and Health Sciences, United Arab Emirates (UAE) University, Al Ain, ARE; 2 Internal Medicine/Rheumatology, Tawam Hospital, Al Ain, ARE; 3 Rheumatology, Sheikh Shakhbout Medical City, Abu Dhabi, ARE; 4 Endocrinology, Tawam Hospital, Al Ain, ARE

This article has been corrected to accurately reflect T-scores and type of score for atherosclerotic cardiovascular disease risk. 

“Bone mineral density (BMD) parameters of GE Lunar dual-energy X-ray absorptiometry (DXA) machine (GE Medical Systems Ultrasound & Primary Care Diagnostics, Madison, Wisconsin, United States) were as follows: 0.751 g/cm2 for L1-4 (T score: 3.6), 0.882 g/cm2 for the right femoral neck (T score: 1.0), 0.874 g/cm2 for the left femoral neck (T score: 1.1), and 0.576 g/cm2 for the left radius 33% (T score: 3.4). Her atherosclerotic cardiovascular disease (ASCVD) risk over the next 10 years is 22.8%.” 

has been changed to 

“Bone mineral density (BMD) parameters of GE Lunar dual-energy X-ray absorptiometry (DXA) machine (GE Medical Systems Ultrasound & Primary Care Diagnostics, Madison, Wisconsin, United States) were as follows: 0.751 g/cm2 for L1-4 (T score: – 3.6), 0.882 g/cm2 for the right femoral neck (T score: – 1.0), 0.874 g/cm2 for the left femoral neck (T score: – 1.1), and 0.576 g/cm2 for the left radius 33% (T score: – 3.4). Her atherosclerotic cardiovascular disease (ASCVD) risk over the next 10 years using the QRISK3 score is 22.8%.”

The authors and journal regret that these errors were not identified and addressed prior to publication.

 

